# Architecture of the caveolar coat complex

**DOI:** 10.1242/jcs.191262

**Published:** 2016-08-15

**Authors:** Alexander Ludwig, Benjamin James Nichols, Sara Sandin

**Affiliations:** 1 School of Biological Sciences, Nanyang Technological University, 60 Nanyang Drive, 637551Singapore; 2 MRC Laboratory of Molecular Biology, Francis Crick Avenue, Cambridge CB2 0QH, UK; 3 NTU Institute of Structural Biology, Nanyang Technological University, Experimental Medicine Building, 59 Nanyang Drive, 637551Singapore

**Keywords:** Caveolae, Caveolar coat, Caveolin, Cavin, Cryo-electron tomography

## Abstract

Caveolae are specialized membrane domains that are crucial for the correct function of endothelial cells, adipocytes and muscle cells. Caveolins and cavins are both required for caveolae formation, and assemble into a large (80S) caveolar coat complex (80S-CCC). The architecture of the 80S-CCC, however, has not been analyzed. Here, we study the 80S-CCC isolated from mammalian cells using negative stain electron microscopy and 3D cryo-electron tomography. We show that the 80S-CCC is a hollow sphere with a diameter of 50–80 nm, and so has the same size and shape as individual caveolar bulbs. This provides strong evidence that the distinctive membrane shape of caveolae is generated by the shape of the 80S-CCC itself. The particle appears to be made up of two layers, an inner coat composed of polygonal units of caveolins that form a polyhedral cage, and an outer filamentous coat composed of cavins. The data suggest that the peripheral cavin coat is aligned along the edges of the inner polyhedral cage, thereby providing a mechanism for the generation of a morphologically stable caveolar coat.

## Abstract (precis)

**Highlighted Article:** This paper reports the first structural analysis of the entire caveolar protein coat isolated intact from mammalian cells, and shows that the coat is composed of two morphologically distinct layers.

## INTRODUCTION

Caveolae are abundant flask- or cup-shaped invaginations in the plasma membrane that are found in almost all vertebrate cells (Stan, 2005). Increasing evidence implicates caveolae in protecting cells from mechanical stress, as well as further potential functions in signaling and membrane homeostasis (Cheng and Nichols, 2016). Caveolae are composed of two protein families, caveolins (caveolin-1, -2, and -3) and cavins (cavin 1–4, also known as PTRF, SDPR, SRBC or PRKCDBP, and MURC, respectively) (Hansen and Nichols, 2010; Kovtun et al., 2015). Caveolin-1 (and caveolin-3 in muscle) and cavin-1 are essential for the formation of caveolae *in vivo* (Drab et al., 2001; Hill et al., 2008; Liu and Pilch, 2008), and mutations in caveolin or cavin genes lead to a variety of human diseases (Ding et al., 2014; Liu et al., 2008; Woodman et al., 2004; Rajab et al., 2010; Hayashi et al., 2009). The characteristic shape of caveolae is likely to be important for caveolar function, but how caveolins and cavins generate the caveolar membrane coat has remained elusive (Shvets et al., 2014).

Caveolae are decorated with a characteristic striated or filamentous coat that wraps all around the caveolar bulb (Peters et al., 1985; Rothberg et al., 1992; Lebbink et al., 2010; Stan, 2005). It was originally suggested that the coat is composed of oligomeric forms of caveolins (Rothberg et al., 1992; Fernandez et al., 2002). The observation that full-length caveolin-1 expressed in bacteria induces the formation of vesicles that resemble native caveolae appears to support this notion (Walser et al., 2012). However, such heterologous (h-)caveolae lack the striated coat and instead exhibit a polyhedral arrangement of caveolins.

It is now clear that cavins are important structural components of caveolae (Gambin et al., 2014; Kovtun et al., 2015, 2014; Ludwig et al., 2013; Shvets et al., 2014; Hansen et al., 2013). Cavins are cytoplasmic proteins that assemble into large homo- and hetero-oligomeric complexes (Bastiani et al., 2009; Hansen and Nichols, 2010; Hayer et al., 2010; Ludwig et al., 2013). All cavins possess two conserved helical regions (HR1 and HR2) and patches of basic residues with affinity to phosphatidylinositol 4,5-bisphosphate [PI(4,5)P_2_] and phosphatidylserine. The N-terminal HR1 domain forms a trimeric coiled-coil that is 2.5 nm wide and 15 nm long (Kovtun et al., 2014). When expressed in bacteria and purified in the presence of detergents, full-length cavins assemble into rod-like structures (Kovtun et al., 2014). These rods might account for the striated appearance of the coat, but this has not been shown directly.

We have recently demonstrated that caveolins and cavins assemble into a distinct 80S particle, which we termed the caveolar coat complex (80S-CCC) (Ludwig et al., 2013). The 80S-CCC contains caveolins and cavins at a defined stoichiometry, and all of its components are distributed all around the caveolar bulb. Whether the 80S-CCC represents an intermediate state in the overall coat, or the entire coat of a single caveolar bulb, is unknown. Here, we set out to study the architecture of the 80S-CCC isolated intact from HeLa cells using negative stain electron microscopy and cryo-electron tomography.

## RESULTS AND DISCUSSION

**Fig. 1.**
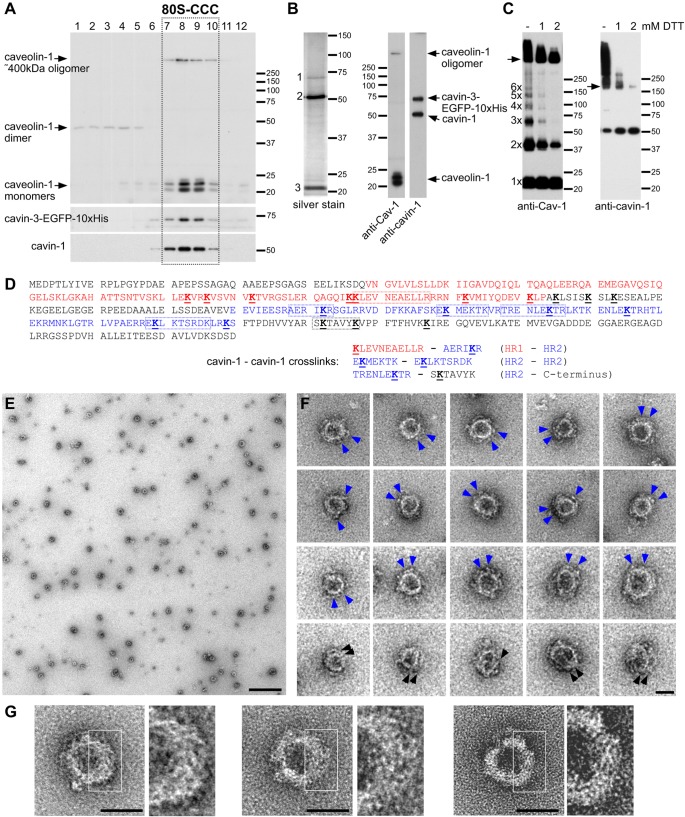
**Purification and negative stain electron microscopy of the caveolar coat complex.** (A) Sucrose gradient fractionation of DSP-crosslinked lysates from HeLa cells stably transfected with a cavin-3–EGFP-10×His protein. Note the discrete peak of the 80S-CCC in fractions 7–10 (boxed). (B) Silver staining (left) and western blots (right) of the purified and fully reduced 80S-CCC. Three proteins are detected by silver staining: cavin-3–EGFP-10×His (1), cavin-1 (2) and caveolins (3). Cav-1, caveolin-1. (C) Partial reduction of DSP crosslinks by titration of DTT. Arrows indicate the ∼400 kDa caveolin-1 oligomer (left) and the ∼180 kDa cavin-1 trimer (right). (D) Protein sequence of human cavin-1 (NP_036364.2) highlighting DSP-modified lysine residues identified by LC-MS/MS. The HR1 domain (amino acids 49–163) is shown in red, the HR2 domain (amino acids 210–300) is shown in blue, modified lysine residues are bold and underlined, and peptides involved in bivalent crosslinks are boxed. Crosslinks between cavin-1 peptides are shown. (E–G) Electron micrographs of purified 80S-CCC in negative stain. (E) Field view. (F,G) Gallery of representative 80S-CCC particles. Blue arrowheads indicate peripheral densities, black arrowheads indicate spirals or filaments in the particle. Scale bars: 500 nm (E); 50 nm (F,G).

In order to isolate the 80S-CCC in its native form, we established a purification protocol that exploited a HeLa cell line expressing cavin-3 fused at its C-terminus to an EGFP-10×His tag (Ludwig et al., 2013). Live HeLa cells were cross-linked with dithiobis(succinimidyl propionate) (DSP), a membrane permeable, reversible, homo-bifunctional crosslinker. The cross-linked 80S-CCC formed a discrete peak in sucrose gradients (Fig. 1A) (Ludwig et al., 2013). The peak fractions 7–10 were pooled and the complex affinity-purified as described in the Materials and Methods. Silver staining and western blotting showed that the complex contained caveolin-1, cavin-1 and cavin-3–EGFP-10×His (Fig. 1B). Cavin-3–EGFP-10×His was the least abundant protein in the complex, which is in agreement with the stoichiometry of the 80S-CCC determined previously (Ludwig et al., 2013). Partial reduction of crosslinks further revealed that the 80S-CCC is composed of ∼400-kDa caveolin-1 oligomers and cavin-1 trimers (Fig. 1C) (Ludwig et al., 2013). In addition, discrete oligomeric forms of caveolin-1 were detected, suggestive of linear growth of caveolin-1 monomers into a large ∼400-kDa particle. Liquid chromatography tandem mass spectrometry (LC-MS/MS) of the isolated complex confirmed the presence of caveolin-1, cavin-1 and cavin-3–EGFP-10×His (not shown). Moreover, we detected DSP-modifications in 19 unique cavin-1 peptides. Seven out of 11 lysine residues in the HR1 domain and six out of 17 lysine residues in the HR2 domain were modified (Fig. 1D). LC-MS/MS of the cross-linked (non-reduced) complex further revealed three distinct cross-links between cavin-1 peptides, all of which involved lysine residues in the HR1 or HR2 domains. No cross-links were detected between cavin-3 and cavin-1 or cavin-1 and caveolin-1 peptides, suggesting that intermolecular cross-links between the cavin-1 HR domains stabilize the 80S-CCC.

To investigate the overall shape and structure of the purified 80S-CCC, we studied the complex by negative stain electron microscopy (Fig. 1E). The complex appeared as a spherical particle with a diameter of 65.9±9.5 nm (mean±s.d.; *n*=243) (Fig. S1). This is consistent with the dimensions of individual caveolae inside cells (Richter et al., 2008) (Fig. S2), implying that the 80S particle represents the entire protein coat of a single caveolar bulb. The particle was composed of a central ring and distinct peripheral densities (Fig. 1F), and at higher magnification, appeared to be composed of a meshwork of fine filaments (Fig. 1G). Images of the 80S-CCC in negative stain therefore show that the particle has the same size and shape as the caveolar bulb, and suggest that the 80S-CCC is composed of two morphologically distinct layers.

**Fig. 2.**
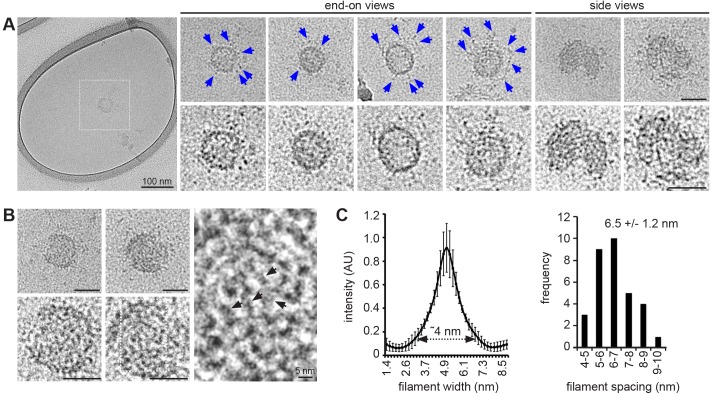
**The caveolar coat complex visualized by electron cryo-microscopy.** (A) Gallery of representative electron micrographs of the 80S-CCC in vitreous ice. The top panels show the particles after gaussian filtering, the bottom row shows close-ups of the same particles after contrast enhancement. Blue arrowheads indicate peripheral densities. (B) Two representative electron micrographs showing the filamentous meshwork in the 80S-CCC. Black arrowheads indicate spirals or filaments in the particle. (C) Quantification of filament width and spacing (mean±s.d., *n*=32 filaments from four particles). Scale bars: 50 nm unless stated otherwise.

Next, we studied the 80S-CCC in vitreous ice by electron cryo-microscopy. As expected, the particle appeared spherical, with a fairly compact central ring and more loosely organized peripheral densities (Fig. 2A). In addition, a zig-zag meshwork of filaments or striations was apparent (Fig. 2B). The filaments had a diameter of ∼4 nm and a mean spacing of 6.5±1.2 nm (mean±s.d.; *n*=32) (Fig. 2C). This is remarkably similar to the dimensions of cavin complexes purified from bacteria and visualized by negative stain electron microscopy (Kovtun et al., 2014). We conclude that the filamentous protein densities observed in negative stain (Fig. 1G) and in ice (Fig. 2C) are likely to be composed of cavin oligomers.

**Fig. 3.**
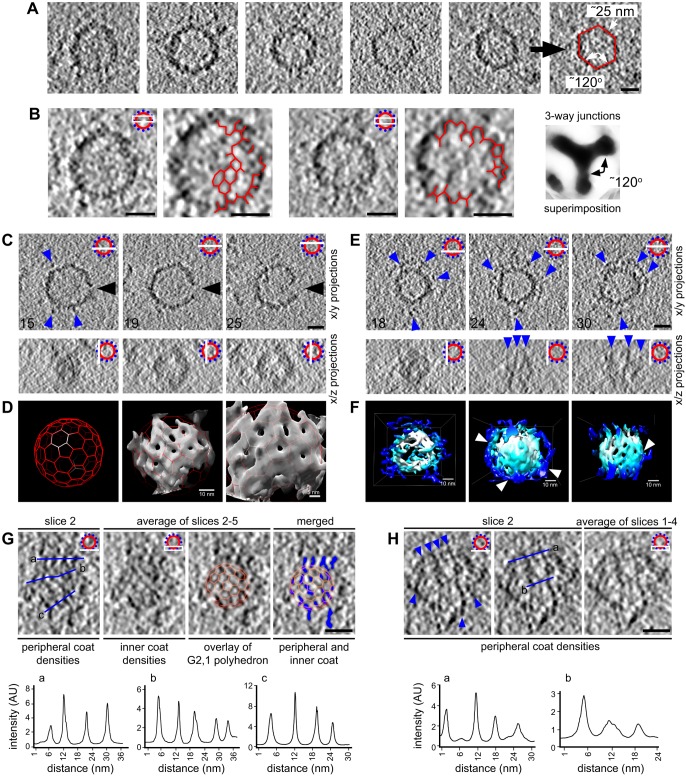
**3D cryo-electron tomography of the caveolar coat complex reveals a two-layered coat architecture.** (A) Gallery of equatorial tomographic slices of five representative particles. Edge lengths and dihedral angles are exemplified for one particle (right). (B) Average intensity projections of tomographic equatorial slices of two particles. Three-way junctions and polygonal profiles are highlighted. The image on the right shows superimposition of 10 three-way junctions. (C,E) Gallery of tomographic slices of two representative particles. Numbers indicate *z*-slice shown. Blue arrowheads depict discrete peripheral densities. The large black arrowhead in C indicates a gap in the protein coat, which is likely to correspond to the opening of the caveolar neck. (D) 3D surface rendering of the particle shown in C. Shown are a G2,1 polyhedral cage (left), the same cage superimposed onto the particle density (middle) and a close-up view (right). (F) 3D surface rendering of the particle shown in E. The central polyhedral cage and the peripheral filamentous coat are colored in white and blue, respectively. Arrowheads point to contacts between the central and peripheral coats. Shown are a top, end-on view (left), a side view (middle) and a sliced side view exposing the inner polyhedral cage (right). (G) Tomographic analysis of the pole of an 80S-CCC. An overlay with a G2,1 polyhedral cage is shown to illustrate the alignment of the peripheral densities (pseudo-colored in blue) along the edges of the inner polyhedral cage. (H) Tomographic analysis of the pole of an 80S-CCC. Blue arrowheads indicate filamentous densities with regular spacing. Scale bars: 20 nm unless stated otherwise. In B, C, E, G and H, a diagram of the slice(s) shown in the image is present at the top right.

In order to study the 3D architecture of the 80S-CCC, we carried out cryo-electron tomography (Fig. 3). Tomography showed that the 80S-CCC is a hollow sphere. Interestingly, rather than adopting a perfectly round or oval shape, the particles often exhibited distinct, albeit rounded, edges and an overall polygonal shape with six roughly planar surfaces. The surfaces were connected at ∼120° angles (119.7±9.4°; mean±s.d.; *n*=6 particles) and had an average edge length of 24.5±3.6 nm (mean±s.d.; *n*=6 particles) (Fig. 3A). Moreover, projections of tomographic slices revealed a partially resolved network of three-way junctions within the 80S-CCC (Fig. 3B). Manual superimposition of multiple junctions confirmed their three-way morphology, and corroborated that their arms were connected by ∼120° angles. The presence of three-way junctions is characteristic of a polygonal or hexagonal arrangement of protein density within the 80S-CCC. Indeed, polygonal profiles could be partially resolved both in tomographic cross-sections (Fig. 3B) and after 3D volume rendering of individual particles (Fig. 3D,F). These observations agree with previous work showing that full-length caveolin-1 expressed in bacteria generates vesicles (h-caveolae) with polyhedral geometry (Ariotti et al., 2015; Walser et al., 2012). We conclude that the 80S-CCC has a roughly polyhedral shape, which might be generated by repeating units of caveolins.

The above data imply that the 80S-CCC confers a polyhedral shape to caveolar membranes. To test this directly, we labeled caveolae *in situ* using APEX2, an engineered ascorbate peroxidase that serves as a genetically encoded reporter for electron microscopy (Lam et al., 2015). Transfection of a caveolin-1–APEX2–EGFP fusion protein into caveolin-1*^−/−^* immortalized mouse embryonic fibroblasts (iMEFs) (which do not have caveolae) rescued caveolae formation (Fig. S2A,B), indicating that the fusion protein is functional. 2D electron microscopy imaging showed that many caveolae indeed had an approximately hexagonal shape (Fig. S2C,D), with edge lengths (31.6±3.9 nm; mean±s.d.; *n*=86) and dihedral angles (123.4±9.7°; mean±s.d.; *n*=67) similar to those observed in the isolated 80S-CCC. Taken together, our data suggest that the caveolar coat possesses polyhedral geometry.

In line with our previous experiments, we found that in ∼20% of particles two layers of density could be resolved (Fig. 3C,E,F; Fig. S3). Projections along the *z*-axis indicated that the more peripheral densities were filamentous (Fig. 3E). 3D volume rendering confirmed this notion and revealed direct contacts between the peripheral and inner layers (Fig. 3F). To investigate the spatial relationship between the two layers in more detail, we analyzed tomographic slices through the poles of the 80S-CCC (Fig. 3G,H). We noticed that the spacing between the filamentous densities was remarkably regular (6.9±1.5 nm; mean±s.d.; *n*=17). This periodicity is in good agreement with the spacing of cavin filaments in our 2D electron cryo-microscopy images (Fig. 2C), as well as with the spacing of cavins *in situ* determined by miniSOG labeling (Ludwig et al., 2013). Averaging of tomographic slices through the pole of the coat (total *z* depth of ∼5 nm) again revealed a partially resolved network of three-way junctions and polygonal densities (Fig. 3G). Overlay of the two layers of densities showed that the peripheral filamentous densities were primarily aligned along the edges of the inner polyhedral cage. We conclude that the peripheral densities are composed of filamentous cavin oligomers, which project along the edges of the inner polyhedral cage.

**Fig. 4.**
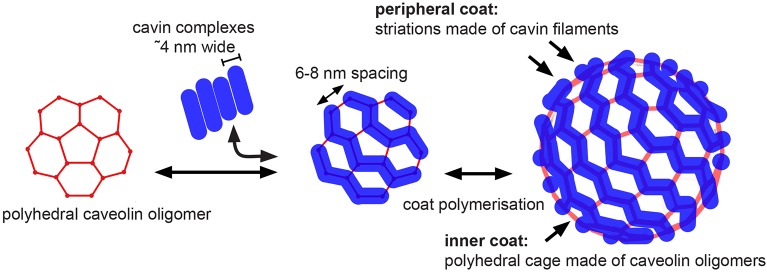
**Model of caveolar coat assembly and architecture.** Caveolins might oligomerize into a polyhedral repeating unit that can further polymerize into different polyhedral cages. Filamentous cavin oligomers associate with the edges of the polyhedral units and with negatively charged membrane lipids, thereby stabilizing the inner polyhedral cage. The peripheral cavin coat produces striations with a periodicity of 6–8 nm.

We show here that the 80S-CCC has the size and shape of the entirety of the distinctive caveolar bulb. Thus, this large, stable protein complex is likely to be the key structural element conferring shape on caveolar membranes. In addition, our data suggest that the 80S-CCC is made of two layers – an inner layer composed of caveolins that assemble into a polyhedral cage, and a peripheral filamentous layer composed of cavins (Fig. 4). A two-layer coat is in line with previous electron microscopy studies of caveolae ultrastructure in ultrathin sections, which revealed intramembrane densities and a sparse spike-like cytoplasmic coat on caveolar membranes (Richter et al., 2008).

Our structural analyses of the isolated 80S-CCC and of caveolae labeled with a caveolin-1–APEX2 fusion protein show that the caveolar coat exhibits features reminiscent of a polygonal cage. Although the edges and surfaces of the protein coat were often rounded or curved, our data confirm, and extend upon, the observation that caveolin-1 expressed in bacteria generates vesicles (h-caveolae) with polyhedral geometry (Ariotti et al., 2015; Walser et al., 2012). We suggest that the caveolar coat adopts an ‘imperfect’ but overall polygonal shape, which is brought about by the geometry of the 80S-CCC and the curvature of the underlying membrane.

We were unable to elucidate the internal architecture of the inner polyhedral cage. This might be due to technical difficulties in fully preserving protein–protein interactions within the particle during isolation. Alternatively, the polyhedral cage might be flexible and/or structurally heterogeneous in nature, and hence challenging to study. Inherent flexibility is somewhat expected given the non-uniform size and shape of caveolae, and analogous architectural flexibility observed in clathrin coats (Cheng et al., 2007), COPI and COPII coats (Faini et al., 2012; Zanetti et al., 2013), and in virus capsids (Schur et al., 2015). Although it is likely that oligomeric forms of caveolins constitute the building blocks of the polyhedral cage (Ariotti et al., 2015; Walser et al., 2012), it is unclear at present how caveolins polymerize into a polyhedron, and whether the polymerized cage is regular or irregular.

In 20% of particles, we observed a second, peripheral, layer of density, which we suggest is composed of cavin filaments. Given that our purification strategy relies on cavin-3–EGFP-10×His to be associated with the 80S-CCC, it is unlikely that the remaining 80% of particles lack the peripheral cavin coat. Instead, we suggest that in the majority of particles the peripheral and inner layers are tightly associated, and thus could not be discriminated at our current resolution. In those cases where two layers could be resolved, we observed that the peripheral cavin filaments were aligned along the edges of the inner polyhedral cage. Such an arrangement of cavins might produce the characteristic striations on the cytoplasmic face of caveolae (Lebbink et al., 2010; Peters et al., 1985; Rothberg et al., 1992; Stan, 2005), stabilize interactions between individual caveolin oligomers, and provide stability to the caveolar coat. Definitive answers to these questions will require higher resolution structural information.

## MATERIALS AND METHODS

### Antibodies, cell lines and cell culture

The following antibodies were used: mouse anti-GFP (1:2000, Roche, Mannheim, 11814460001), rabbit anti-PTRF (cavin-1; 1:2000, Abcam, Cambridge, ab48824), and rabbit anti-caveolin-1 (1:10,000, BD Biosciences, 610060). The clonal HeLa cell line stably expressing the cavin-3–EGFP-10×His protein has been described previously (Ludwig et al., 2013). Cells were cultured in Dulbecco's modified Eagle's medium (DMEM), 10% fetal calf serum (FCS), penicillin-streptomycin (LifeTechnology, Singapore) and 0.2 mg/ml G418 (Sigma, Singapore) at 37°C and under a 5% CO_2_ atmosphere.

### Purification of the 80S-CCC

A total of 20 150-mm dishes of confluent cultures of HeLa cells were cross-linked with 2 mM DSP (LifeTechnology, Singapore) as described previously (Ludwig et al., 2013). Cells were scraped into 0.8 ml of lysis buffer per dish [50 mM Tris-HCl pH 8, 300 mM NaCl, 0.5% (v/v) Triton X-100, 1% (w/v) octyl-glucoside and protease inhibitor cocktail (Roche, Mannheim)] and cleared by centrifugation (20,000 ***g*** for 30 min). Lysates were added on top of a linear 20–40% (w/v) sucrose gradient prepared in 50 mM Tris-HCl pH 8, 300 mM NaCl and 0.2% Triton X-100. One gradient contained 3 ml of each 40%, 30% and 20% sucrose and was overlaid with 3 ml of lysate. Gradients were spun in a SW41Ti rotor at 234,745 ***g*** max for 6 h at 4°C and 12 1-ml fractions were collected. The peak fractions 7–10 were pooled and diluted 1:1 with 50 mM Tris-HCl pH 8, 300 mM NaCl and 20 mM imidazole. This was incubated with 500 µl TALON metal affinity resin (Clontech, Singapore) for 4 h at 4°C. The suspension was applied to 6-ml prep columns, washed three times with 6 ml of 50 mM Tris-HCl pH 8, 300 mM NaCl and 20 mM imidazole, and eluted with 50 mM Tris-HCl pH 8, 150 mM NaCl and 400 mM imidazole. Four 200-µl elution fractions were collected. The complex eluted sharply in fractions 2 and 3. Fraction 2 had a protein concentration of 20–50 ng/µl [as estimated by silver staining (Fig. 1B)], and was used undiluted for all further analyses.

### Negative stain electron microscopy

5 µl of freshly purified 80S-CCC was deposited on continuous carbon-coated 300 mesh copper grids, washed with three drops of water, and negatively stained with 4 µl of 0.2–1% uranyl acetate. Electron micrographs were recorded on a Tecnai T12 (FEI) transmission electron microscope (TEM) operated at 120 kV using a 4k×4k Eagle (FEI Company) CCD camera and a defocus range of −1 to −4 µm.

### Electron cryo-microscopy

For electron cryo-microscopy and tomographic analysis, 10 µl of freshly purified 80S-CCC was applied to glow-discharged 200 mesh Quantifoil R2/2, holey or lacey copper electron microscopy grids coated with a 10 nm layer of carbon. For tomography, 10-nm BSA-coated gold particles (BBI) were applied to the elution fractions prior to application to the grids. Cryo grids were prepared with a Vitrobot (FEI Company) plunger using liquid ethane as the freezing agent. Micrographs were recorded on a Tecnai Arctica (FEI Company) operated at 200 kV, using a Falcon II (FEI Company) direct electron detector. 2D images were recorded at underfocus (−2 to −5 µm), a nominal magnification of 53,000× (corresponding to an object pixel size of 2 Å), and an electron dose of 30 e/Å^2^. Single-axis tilt series were recorded at +/−65°, recording an image at 2° intervals, using low-dose data acquisition routines (Tomo FEI). The total dose per tilt-series was 60 e/Å^2^. The nominal magnification was 23,000×, corresponding to an object pixel size of 4.8 Å. Tilt series were binned by a factor of two and reconstructed into 3D tomograms by filtered back-projection (Crowther and Klug, 1975) using the IMOD software package (Kremer et al., 1996).

### Image analysis

Approximately 100 2D electron cryo-microscopy images and 20 cryo-electron tomograms were used for analysis. The architecture of 50–60 reconstructed particles was analyzed in depth and ten of those were used for 3D volume rendering in Chimera software (Pettersen et al., 2004). Image analysis and line-scans were carried out in ImageJ or Fiji software (Schindelin et al., 2012; Schneider et al., 2012). 2D electron cryo-microscopy images were de-noised by applying a mild gaussian filter (two pixels) and corrected for brightness and contrast for better visualization.

### APEX2 labeling for electron microscopy

Immortalized mouse embryonic fibroblasts (iMEFs) from caveolin-1^−/−^ mice (Hansen et al., 2013) were grown on fibronectin-coated (Sigma) glass-bottom dishes (MatTec Corp., Ashland, MA) and transfected with 1.5 µg caveolin-1–APEX2–EGFP plasmid DNA using FugeneHD (Promega, Singapore). At 48 h post transfection, cells were fixed with 2% glutaraldehyde (EMS, Hatfield, PA) and 2 mM CaCl_2_ in 0.1 M cacodylate buffer pH 7.4 (EMS) for 1 h on ice and further processed for APEX labeling and electron microscopy (Lam et al., 2015). Electron micrographs were recorded on a Tecnai T12 (FEI Company) TEM operated at 120 kV using a 4k×4k Eagle (FEI) CCD camera.
